# Geographical heterogeneity and inequality of access to improved drinking water supply and sanitation in Nepal

**DOI:** 10.1186/s12939-018-0754-8

**Published:** 2018-04-02

**Authors:** Wen-Jun He, Ying-Si Lai, Biraj M. Karmacharya, Bo-Feng Dai, Yuan-Tao Hao, Dong Roman Xu

**Affiliations:** 1 Department of Medical Statistics and Epidemiology, School of Public Health, Sun Yat-sen University, Guangzhou, 510080 China; 2 Division of Cardiology, University of Washington, Seattle, WA USA; 3 Department of Global Health, University of Washington, Seattle, WA USA; 4 Department of Community Programs, Kathmandu University School of Medical Sciences, Dhulikhel, Nepal; 5 Sun Yat-sen Global Health Institute, Sun Yat-sen University, Guangzhou, 510275 China; 6 Guangdong Key Laboratory of Health Informatics, Health Information Research Center, Department of Medical Statistics and Epidemiology, School of Public Health, Sun Yat-sen University, Guangzhou, 510080 Guangdong China

**Keywords:** Sanitation, Drinking water, Heterogeneity, Inequality, Nepal

## Abstract

**Background:**

Per United Nations’ Sustainable Development Goals, Nepal is aspiring to achieve universal and equitable access to safe and affordable drinking water and provide access to adequate and equitable sanitation for all by 2030. For these goals to be accomplished, it is important to understand the country’s geographical heterogeneity and inequality of access to its drinking-water supply and sanitation (WSS) so that resource allocation and disease control can be optimized. We aimed 1) to estimate spatial heterogeneity of access to improved WSS among the overall Nepalese population at a high resolution; 2) to explore inequality within and between relevant Nepalese administrative levels; and 3) to identify the specific administrative areas in greatest need of policy attention.

**Methods:**

We extracted cluster-sample data on the use of the water supply and sanitation that included 10,826 surveyed households from the 2011 Nepal Demographic and Health Survey, then used a Gaussian kernel density estimation with adaptive bandwidths to estimate the distribution of access to improved WSS conditions over a grid at 1 × 1 km. The Gini coefficient was calculated for the measurement of inequality in the distribution of improved WSS; the Theil *L* measure and Theil *T* index were applied to account for the decomposition of inequality.

**Results:**

57% of Nepalese had access to improved sanitation (range: 18.1% in Mahottari to 100% in Kathmandu) and 92% to drinking-water (range: 41.7% in Doti to 100% in Bara). The most unequal districts in Gini coefficient among improved sanitation were Saptari, Sindhuli, Banke, Bajura and Achham (range: 0.276 to 0.316); and Sankhuwasabha, Arghakhanchi, Gulmi, Bhojpur, Kathmandu (range: 0.110 to 0.137) among improved drinking-water. Both the Theil *L* and Theil *T* showed that within-province inequality was substantially greater than between-province inequality; while within-district inequality was less than between-district inequality. The inequality of several districts was higher than what is calculated by regression of the Gini coefficient and our estimates.

**Conclusions:**

This study showed considerable geographical heterogeneity and inequality not evidenced in previous national statistics. Our findings may be useful in prioritizing resources to reduce inequality and expand the coverage of improved water supply and sanitation in Nepal.

## Background

Access to a safe drinking-water supply and good sanitation (WSS) is considered a basic human right [1] as it is crucial not only for the health of an individual and a population but also for the quality of life and dignity of both. Sufficient, safe, acceptable, physically accessible and affordable water and sanitation reduce a wide range of health risks, especially those associated with water-borne diseases including diarrhea, schistosomiasis and neglected tropical diseases (NTDs) [2–7].

Defined by Millenium Development Goals (MDGs) of the United Nations (UN), improved WSS comprise sanitation facilities that hygienically isolate human excreta from human contact and drinking-water sources that guard against outside contamination. Remarkable progress to access to WSS has been made with regard to MDGs [8], which targeted reducing the population without access to improved drinking water and basic sanitation by half. Reports from the WHO and UNICEF’s Joint Monitoring Program for Water Supply and Sanitation (JMP) announced that the target for achieving safe drinking water was met globally in 2010, well ahead of the MDG 2015 deadline [8]. In general, 2.6 billion people had gained access to an improved drinking-water source and 2.1 billion to improved sanitation between 1990 and 2015. However, the MDG target for sanitation was missed by nearly 700 million people, leaving a total of 2.4 billion globally still without access to sanitation facilities in 2015 [8]. In addition, global and national studies also suggested inequalities in access to WSS between socioeconomic castes and between rural and urban zones [9–13]. As declared in the Assessment of Sanitation and Drinking Water Report [12], wide disparities in access to water and sanitation had become a major challenge in extending and sustaining current services. The inequalities in a region include social, environmental and health inequalities [13]. The spatial inequality of distribution is a problem hidden by regional averages. It is reported that the burden of life lost was twice what it would be if access to improved WSS was fairly distributed [9]. Even during the twenty-first century, 716 million people, including women and children, defecate in the open every day in South Asia, a region known for poverty and poor hygiene but also for rapid economic growth [10], a disparity which has inspired global and national studies suggesting that equity be woven into every investment, supervisory mission and audit [10, 13].

Bordering China to the north and India to the south, Nepal has a population of 26.4 million and a land area of 147,181 km^2^ in 2011 [14]. As a developing nation, Nepal ranked 144th in the Human Development Index in 2016. Approximately 83% of the Nepalese still live in rural areas, and the country’s Gross Domestic Product (GDP) in USD during 2012 was $18.96 billion [15, 16]. As one of the least developed countries in the world, Nepal faces a plethora of problems regarding its drinking-water supply and sanitation [17]. JMP reported that 92% of the Nepalese population in 2015 had access to improved water, which increased by 26% from 1990, and met MDG targets [18]. With good progress but not meeting the target, the coverage of improved sanitation in 2015 was 46%, while 37% of the population still relied on open defecation, still creating a high risk for environmental contamination [18]. A survey from the Department of Health Services of Nepal indicated that approximately 3500 children die of water-borne diseases every year [19]. Having an unprotected water source and poor sanitation are the major contributing factors that lead to a greater risk of acquiring diarrheal disease, which can worsen the burden of disease in Nepal [19]. A better understanding of geographical heterogeneity and inequality of access to improved WSS is important for resource allocation as well as prioritization of the programs that are focused on preventing epidemics [19–21]. Although efforts have been made to estimate the cross-national geographical distribution of WSS [18], to our knowledge no studies have aimed to develop a high-resolution map to critically identify the areas of greatest need in Nepal. High resolution is important and unique in this study as a greater disaggregation in the resultant datasets is necessary for better decision-making, which can be of extreme importance in directing resources and technologies to the specific areas of greatest need. Further, geographical heterogeneity and the coverage of improved WSS is an important factor in dealing with the propagation and spread of neglected tropical diseases (NTDs). As a disease positive case is mainly a point on a map, having a high-resolution version of Nepal’s map will allow us to highlight any direct correspondence between a positive case and the local quality of WSS service, providing an important data source for geostatistical analysis of that relationship.

An in-depth understanding of access to improved WSS service across Nepal is important for policymakers as they focus on achieving the Sustainable Development Goals (SDGs), including universal and equitable access to safe and affordable drinking water and sanitation for all by 2030. As most research on the subject solely describes raw coverage for specific districts [22], or access across the entire nation [8], a higher disaggregation of special distribution is needed. Research to improve coverage and the equity of WSS access is particularly important because it concerns a basic human right and aids in combating the spread of water-borne disease in a highly vulnerable population. Therefore, this study aimed to explore heterogeneity and inequality in access to improved WSS across Nepal by map and administrative levels to prioritize resource allocation and policy-making. The specific goals of this paper are 1) to estimate the spatial heterogeneity of coverage in improved WSS among the overall Nepalese population in a higher disaggregation level and at different administrative levels of Nepal’s government; 2) to explore inequality within and between these administrative levels; and 3) to identify the specific administrative area of greatest need for policy attention (whereinequality is higher than expected or both sanitation and drinking-water supply are lower than the national average).

## Methods

### Study area

Following the new Constitution of Nepal in March 2017, Nepal has been re-arranged into 7 provinces and 75 districts. From the highest to the lowest, administrative levels are divided into provinces, districts, and village development committees [23]. It should be noted that the districts of Nawalparasi and Rukum were administratively designed under two provinces according to the new schedule [23], while in this study, Nawalparasi and Rukum districts are assigned to Province No. 5 and No. 6 respectively.

According to the ecological characteristics of Nepal [24], there are three clear physiographic areas: mountains, hills and the Terai region from north to south. Furthermore, these three ecological belts running from west to east are vertically intersected by a north to south flowing river system. Moreover, Province No. 2 is the most densely populated province in Nepal, consisting mostly of the Terai grasslands, while Provinces No. 6, No. 4 and No. 7 are sparsely populated and located in the mountainous regions. The city of Kathmandu, located in the district of Kathmandu, is the capital of Nepal and both the largest and most densely populated city. The Kathmandu district is located in Province No. 3, which is the most urbanized province [25].

### Data sources

This study required data representing the general situation of WSS as well as the distribution of the population across Nepal. Cluster-sample data from the 2011 Nepal Demographic and Health Survey (DHS) provided the information we required [26]. The survey was based on probability sampling and data collection and was done at household level by trained investigators. The cluster was identified by the GPS coordinates of the centroid of the surveyed households. To ensure the privacy of those surveyed, the coordinates of each cluster were randomly displaced by up to 2 km for urban areas and 5 km for rural areas, respectively, with 1% of rural clusters being offset by up to 10 km. In 2011, the 289 Nepal DHS clusters included 10,826 surveyed households, with 194 rural clusters and 95 urban clusters. The digitally-gridded population density of Nepal in 2011 was obtained from the *WorldPop* project [27], which provided an estimate of the population density for every low and middle income country, plotting it at a high resolution in 100 m by 100 m squares.

For MDG monitoring [28], an improved sanitation facility was described as “hygienically separating human excreta from human contact”, while an improved drinking-water source was defined as being “protected from outside contamination (especially fecal contamination).” Furthermore, JMP defined four types of facilities as improved ((1) Flush or pour-flush to piped sewer system/ septic tank/ pit latrine, (2) Ventilated improved pit (VIP) latrine, (3) Pit latrine with slab, (4) Composting toilet) and six types of drinking-water source ((1) Piped water into dwelling, yard or plot, (2) Public tap or standpipe, (3) Tubewell or borehole, (4) Protected spring, (5) Protected dug well, (6) Rainwater collection [29]).

### Indicators measuring heterogeneity and inequality

The percentage of households defined as possessing improved WSS standards, or coverage of improved WSS access as defined below, was calculated with the kernel density estimation model and plotted across a grid of 1 × 1 km squares [30, 31]. Moreover, estimated coverage at a high resolution is also necessary to evaluate inequality when population density was considered.

Techniques developed for the measurement and decomposition of income inequality are generally appropriate for the distribution of health matters [32]. For measuring inequalities in the distribution of access to improved WSS, two indices including the Theil *L* measure, Theil *T* index [33] and Gini coefficient [34] were calculated. Our reason for choosing these indices is that the Theil *L* and Theil *T* are the most desirable decomposition measures of inequality and the Gini coefficient is the most well-recognized measure [32, 35]. Decomposition means a calculation of two separate components of the whole inequality: the “within-group” and “between-group”. The Theil *L* measure is decomposable in a better sense than the Theil *T*, but the Theil *T* index is complementary to the Theil *L* index when there is zero population in a unit. The Gini coefficient was calculated depending on the deviation of the Lorenz curve from the diagonal line, with 0 representing perfect equality and 1 representing total inequality [32]. For the Gini coefficient, a range from 0 to 0.3 means comparatively fair and 0.4 is considered to be a warning limit; greater than 0.4 indicates the existence of inequality.

Different from income, coverage of improved WSS is defined as the bounded variable in a range of 0–1. Greater coverage means the lower Gini coefficient. The correlation between the two at district level was revealed by Pearson correlation analysis. Furthermore, outlier districts, higher or lower levels of Gini when giving the levels of coverage, were also analyzed by the linear regression between Gini and coverage. The relative geographical inequality index (RGI), developed by Rachel L.Pullan [36], was calculated as the difference between observed and expected Gini,which helped identify the relative unequal districts.

### Data analysis

The kernel density estimation with adaptive bandwidths was employed to estimate coverage of improved WSS in areas without DHS surveys [30]. Instead of using fixed bandwidths, we used adaptive bandwidths which is more flexible and more natural for estimation of spatial relative risk [37]. The Gaussian kernel was applied for surface computing and the adaptive bandwidth was based on the minimal number of observations *N*. With the parameter *N*, the bandwidth of the Gaussian kernel depends on the proportion of a weighted number of households in the cluster. The optimal value of parameter *N* (denoted *No*) was calculated according to the following formula [30]:

$$ No= 1417{2}^{\ast }{n}^{\ast } 0.41{9}^{\ast }p- 0.36{1}^{\ast }{g}^{\ast } 0.037- 91.011 $$

(*p:* observed national prevalence*; n:* the number of persons tested*; g:* the number of clusters surveys)

The estimated coverage of improved WSS was calculated as the ratio between the intensity of the numbers of improved households and total households. The estimation was undertaken using the homonymous R package prevR [30, 31].

To improve the accuracy of our analysis, population data and estimated results were adjusted with other known data [38]. Population density in 2011 Nepal from the *WorldPop* project was not adjusted by a UN estimate. Therefore the population data (*pop*_*adj*_)in our analysis was rescaled to be consistent with the UN’s estimation. As was previously described, we estimated the unadjusted coverage of access to improved WSS (*Coverage*_*unadj*_) at a resolution of 1 × 1 km. Furthermore, the unadjusted number of persons living with improved WSS (*nplwss*_*unadj*_) was calculated as *Coverage*_*unadj ×*_*pop*_*adj*_*.* To make the total number of people living with improved WSS equal to the estimation from JMP, *nplwss*_*unadj*_ was rescaled into *nplwss*_*adj*_. Moreover, the adjusted coverage of access to improved WSS (*Coverage*_*adj*_) was equal to *nplwss*_*adj*_/ *pop*_*adj*_ [38].

All analysis was performed using R 3.1.0 software with the foreign, maptools, raster, prevR, and ineq packages (http://mirrors.ustc.edu.cn/CRAN/).

## Results

### Heterogeneity of coverage in improved WSS

**Fig. 1:**
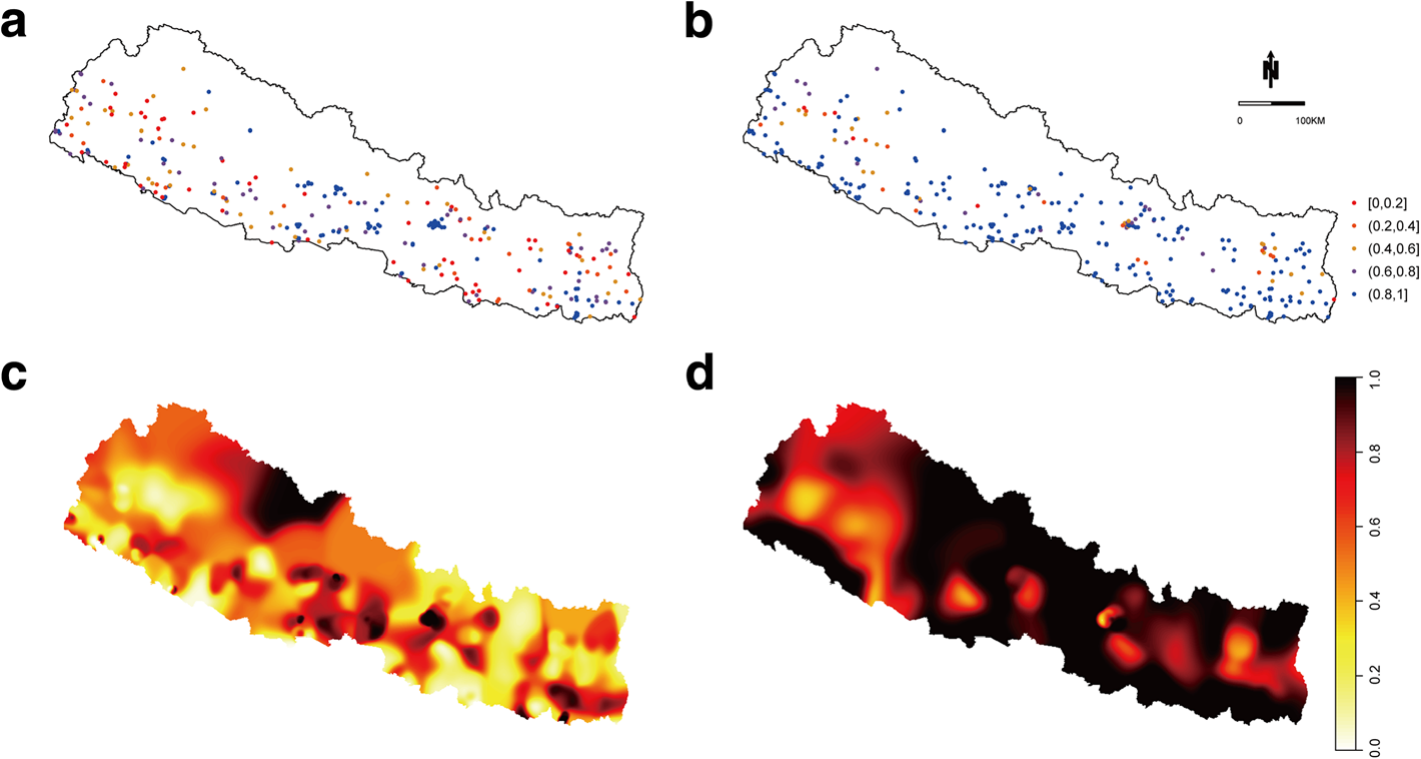
Samples of nationally representative cluster survey on improved WSS and the predicted population coverage in Nepal 2011. (**a**) and (**b**) represent the access to improved sanitation and improved drinking-water supply, respectively, with the same location but different coverage. Predicted improved sanitation at 1 KM^2^ resolution is shown in (**c**) and improved drinking water in (**d**).(The number in the legend is the value of coverage)

A total of 289 survey clusters including 10,826 households were obtained from the 2011 Nepal DHS. The coverage of improved WSS in each cluster is shown in Fig. 1a and b. Sample locations in Fig. 1 emphasize that sampled clusters mostly were located on southern and lowland plains, and access to improved drinking water was in the range of 0.8 to 1.0, higher than the percentage rate of access to improved sanitation. In the kernel density estimation with adaptive bandwidth implemented in prevR packages, the optimal *N* parameter for improved sanitation analysis was 129, while it was 218 for improved drinking water. Figure 1c and d present the coverage of improved sanitation and improved drinking water respectively. Low coverage of both improved sanitation and improved drinking water was mainly observed in the center of northwest Nepal. Southeast Nepal was also relatively weak in the quality of WSS standards, especially regarding sanitation.

**Fig. 2:**
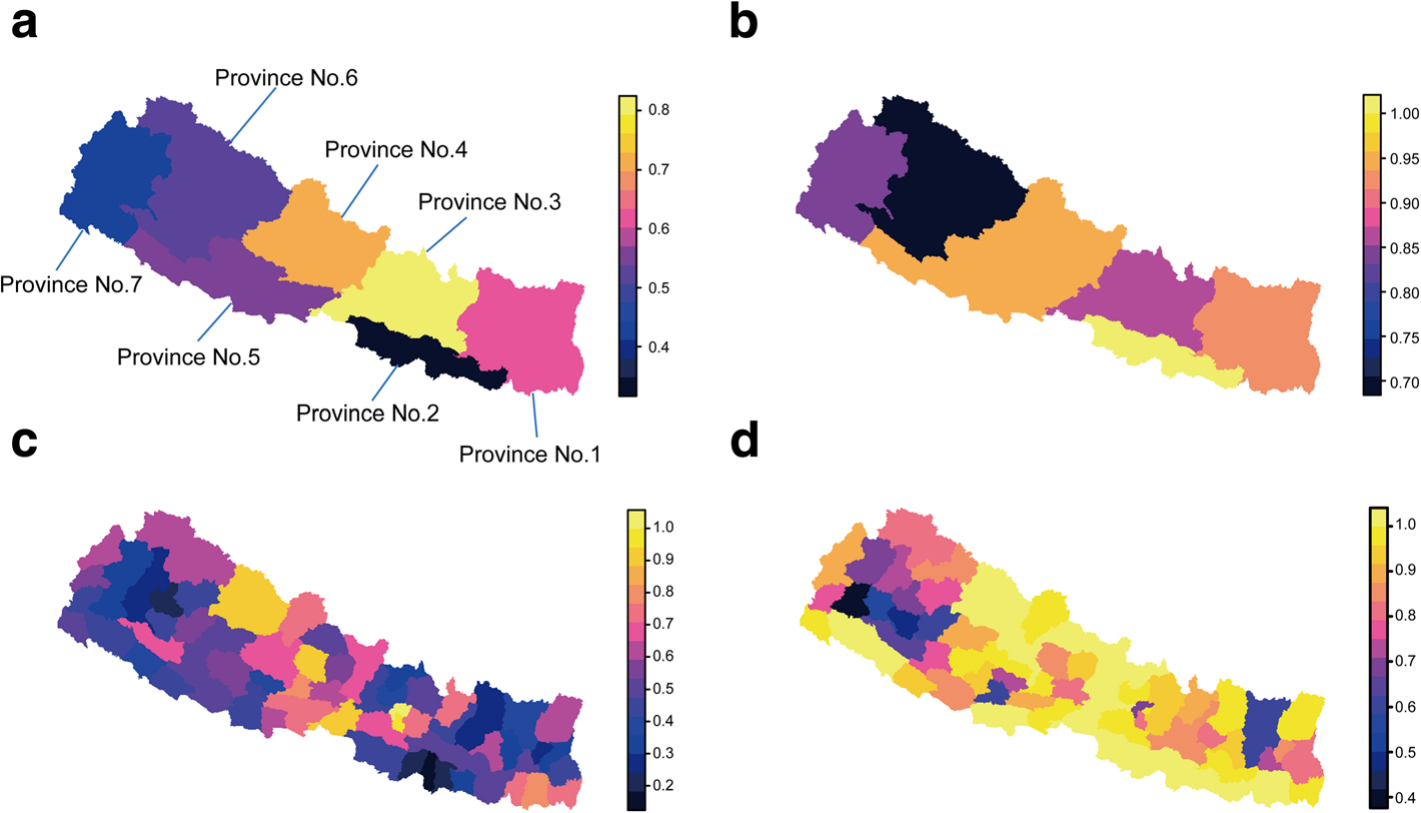
Predicted population coverage presented in provinces and districts. **a** Predicted population coverage of access to improved sanitation in provinces, and (**b**) Predicted population coverage of improved drinking water in provinces. **c** Predicted population coverage of access to improved sanitation in districts, and **d** Predicted population coverage of access to improved sanitation in districts

**Fig. 3:**
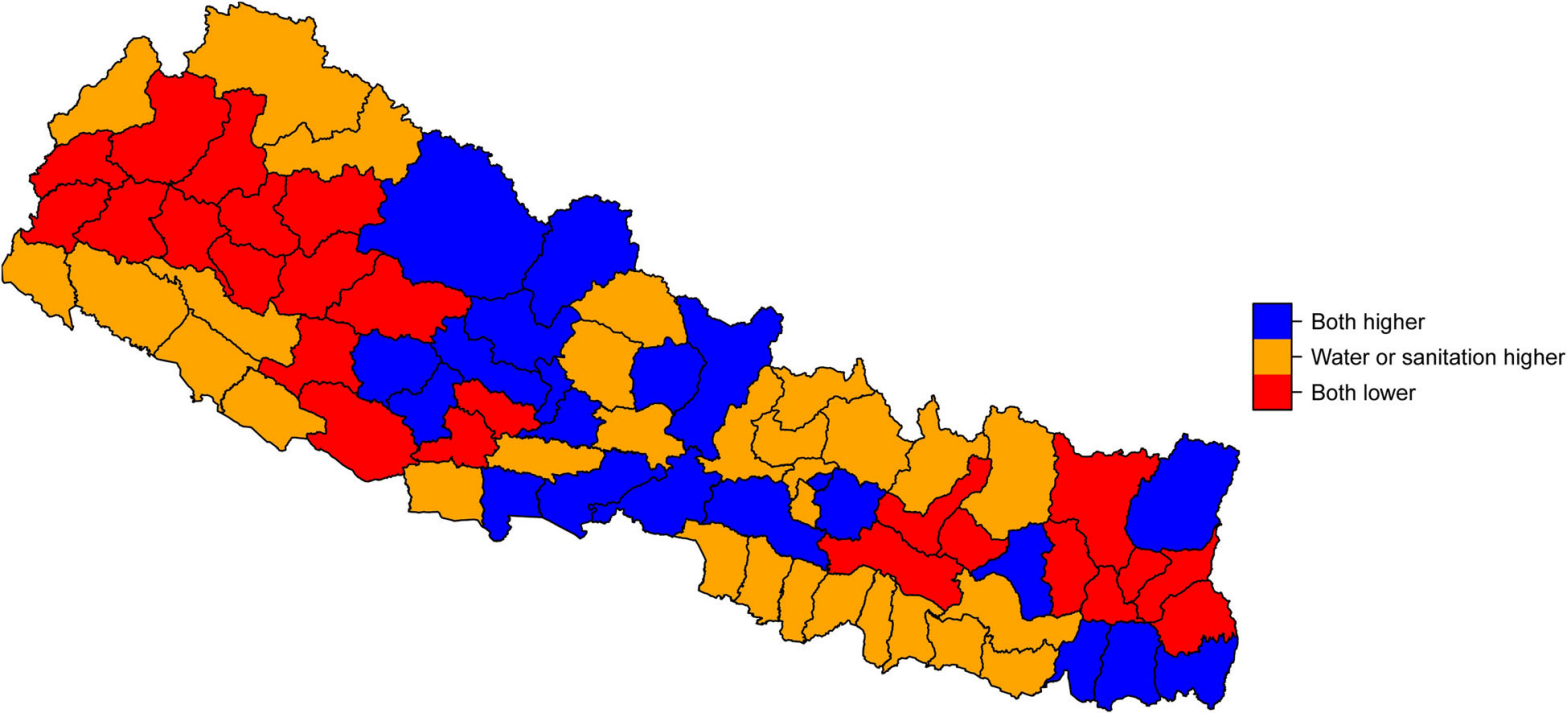
Condition of predicted population coverage of improved WSS in districts compared with national mean coverage

Figure 2 provides the predicted estimate of geographical heterogeneity by province and district; and the details are shown in Tables 1, 2 and 3. The model estimated coverage in country/provinces/districts was mostly close to raw data. Estimations of national coverage were 55.14% for improved sanitation and 87.61% for improved drinking water, which were similar to the JMP estimation of 57% and 92%. We discovered that despite a reported national mean of 92% the distribution of access to improved WSS conditions was greatly unequal across provinces. For example, we estimated 70.5% access to improved drinking water in Province No. 6, while Province No. 2 had 100% access. An even higher disparity was observed with regard to improved sanitation, with Province No. 2 having 34.7% total coverage compared to Province No. 3’s 79.3%. Geographical heterogeneity was more distinct at the district level. Concerning improved sanitation, coverage varied from less than 30% for 9 districts (Okhaldhunga, Terhathum, Bajura, Achham, Solukhumbu, Dhanusa, Kalikot, Sarlahi, Mahottari) to greater than 70% for 15 districts (Kathmandu, Bhaktapur, Lalitpur, Dolpa, Chitawan, Kaski, Morang, Syangja, Jhapa, Sunsari, Palpa, Kavrepalanchok, Nawalparasi, Mustang, Dolakha). Availability of improved drinking water in Dailekh and Doti was less than 50% despite the national coverage of 92%. Figure 3 shows the coverage of improved WSS in districts relative to national coverage: 24 districts were lower than the national mean in improved WSS; these were located mainly in northwest and southeast Nepal.

**Table 1:** Administrative coverages for access to improved WSS and summaries of inequity coefficient

Administrative Region	Improved Indicator	Model Estimate(JMP adj.)	Raw coverage	Population(UN adj.)	Gini	Theil *L*	Theil *T*
Country	Sanitation	0.57	0.584	27,156,000	0.272	0.143	0.119
	Drinking water	0.92	0.858		0.078	0.017	0.015
Province No.1	Sanitation	0.621	0.573	4,654,292	0.227	0.099	0.085
	Drinking water	0.935	0.834		0.073	0.013	0.012
Province No.2	Sanitation	0.347	0.394	5,551,672	0.319	0.167	0.159
	Drinking water	1.00	1.00		0.01	0.001	0.001
Province No.3	Sanitation	0.793	0.709	5,633,266	0.176	0.074	0.06
	Drinking water	0.865	0.841		0.101	0.021	0.018
Province No.4	Sanitation	0.719	0.784	2,164,437	0.145	0.039	0.035
	Drinking water	0.938	0.925		0.062	0.007	0.007
Province No.5	Sanitation	0.541	0.606	4,870,079	0.218	0.098	0.08
	Drinking water	0.942	0.925		0.071	0.014	0.012
Province No.6	Sanitation	0.525	0.525	1,672,443	0.184	0.075	0.062
	Drinking water	0.705	0.656		0.132	0.026	0.026
Province No.7	Sanitation	0.43	0.474	2,609,811	0.263	0.124	0.109
	Drinking water	0.849	0.820		0.136	0.04	0.035

**Table 2:** District estimates for access to improved sanitation and summaries of inequity coefficient

Districts	Model Estimate(JMP adj)	Raw coverage^a^	Gini	RGI score^b^	Theil *L*	Theil *T*
Kathmandu	1.000	0.983	0.004	↓-0.043	0.000	0.000
Bhaktapur	1.000	0.963	0.006	↓-0.045	0.001	0.001
Lalitpur	0.989	1.00	0.026	− 0.025	0.003	0.002
Dolpa	0.885	0.973	0.061	−0.010	0.007	0.007
Chitawan	0.885	0.903	0.047	−0.023	0.005	0.005
Kaski	0.884	0.946	0.083	0.012	0.015	0.014
Morang	0.811	0.854	0.114	↑0.029	0.024	0.022
Syangja	0.769	0.839	0.061	↓-0.032	0.008	0.008
Jhapa	0.747	0.623	0.088	−0.009	0.030	0.022
Sunsari	0.738	0.818	0.163	↑0.065	0.068	0.056
Palpa	0.725	0.795	0.081	−0.019	0.016	0.014
Kavrepalanchok	0.721	0.698	0.126	↑0.024	0.031	0.027
Nawalparasi	0.711	0.779	0.123	0.020	0.035	0.029
Mustang	0.710	–	0.110	0.007	0.020	0.021
Dolakha	0.709	0.703	0.110	0.006	0.023	0.021
Myagdi	0.687	0.561	0.088	−0.019	0.012	0.013
Surkhet	0.684	0.739	0.064	↓-0.044	0.006	0.006
Gorkha	0.678	0.726	0.090	↓-0.019	0.013	0.012
Makwanpur	0.674	0.609	0.129	0.019	0.042	0.034
Baglung	0.674	0.754	0.192	↑0.081	0.082	0.067
Parbat	0.665	0.846	0.184	↑0.072	0.073	0.063
Humla	0.637	0.561	0.041	↓-0.076	0.003	0.003
Mugu	0.636	0.886	0.073	↓-0.044	0.008	0.008
Khotang	0.629	0.753	0.111	−0.007	0.023	0.021
Taplejung	0.612	0.632	0.094	↓-0.028	0.017	0.016
Darchula	0.608	0.651	0.050	↓-0.073	0.005	0.005
Tanahu	0.604	0.671	0.147	↑0.024	0.034	0.033
Rupandehi	0.603	0.753	0.249	↑0.126	0.178	0.121
Lamjung	0.590	0.512	0.060	↓-0.065	0.006	0.006
Pyuthan	0.587	0.746	0.135	0.008	0.050	0.040
Rolpa	0.585	0.351	0.194	↑0.067	0.082	0.066
Jajarkot	0.565	0.538	0.025	↓-0.105	0.001	0.001
Baitadi	0.551	0.592	0.070	↓-0.063	0.013	0.011
Manang	0.530	–	0.001	↓-0.136	0.000	0.000
Udayapur	0.522	0.553	0.229	↑0.091	0.097	0.085
Saptari	0.520	0.457	0.316	↑0.178	0.211	0.167
Salyan	0.520	0.400	0.111	↓-0.028	0.027	0.023
Dang	0.518	0.570	0.163	↓0.024	0.054	0.046
Rukum	0.516	0.470	0.054	↓-0.085	0.009	0.008
Sindhuli	0.508	0.474	0.290	↑0.149	0.162	0.137
Dhankuta	0.499	0.770	0.139	−0.004	0.031	0.030
Kanchanpur	0.492	0.576	0.246	↑0.102	0.126	0.101
Sindhupalchok	0.488	0.450	0.092	↓-0.052	0.014	0.014
Bara	0.470	0.479	0.220	↑0.072	0.101	0.083
Dadeldhura	0.464	0.429	0.099	↓-0.050	0.021	0.019
Kailali	0.463	0.577	0.276	↑0.126	0.128	0.118
Dhading	0.460	0.354	0.163	0.013	0.043	0.043
Parsa	0.458	0.619	0.247	↑0.097	0.128	0.107
Ilam	0.453	0.448	0.182	↑0.031	0.052	0.052
Rautahat	0.452	0.676	0.173	↑0.022	0.051	0.047
Arghakhanchi	0.448	0.380	0.063	↓-0.089	0.006	0.006
Ramechhap	0.447	0.209	0.142	−0.010	0.033	0.032
Kapilbastu	0.442	0.426	0.136	↓-0.017	0.035	0.031
Banke	0.440	0.564	0.284	↑0.130	0.185	0.141
Dailekh	0.420	0.362	0.218	↑0.061	0.081	0.074
Jumla	0.420	0.288	0.136	↓-0.021	0.029	0.029
Gulmi	0.411	–	0.102	↓-0.057	0.018	0.019
Bardiya	0.405	0.363	0.127	↓-0.033	0.030	0.029
Nuwakot	0.388	–	0.145	−0.019	0.035	0.038
Sankhuwasabha	0.369	0.463	0.140	↓-0.027	0.030	0.031
Siraha	0.352	0.320	0.041	↓-0.129	0.004	0.004
Panchthar	0.343	0.250	0.107	↓-0.065	0.019	0.019
Bhojpur	0.330	0.090	0.246	↑0.072	0.092	0.096
Bajhang	0.327	0.364	0.121	↓-0.054	0.022	0.023
Rasuwa	0.325	0.208	0.078	↓-0.097	0.009	0.009
Doti	0.301	0.313	0.239	↑0.060	0.093	0.089
Okhaldhunga	0.288	0.227	0.090	↓-0.092	0.014	0.015
Terhathum	0.280	0.250	0.093	↓-0.091	0.013	0.013
Bajura	0.274	0.247	0.279	↑0.094	0.125	0.121
Achham	0.267	0.258	0.276	↑0.090	0.119	0.118
Solukhumbu	0.243	0.090	0.206	0.015	0.065	0.068
Dhanusa	0.205	0.243	0.201	0.003	0.064	0.063
Kalikot	0.195	0.136	0.165	↓-0.034	0.045	0.050
Sarlahi	0.186	0.160	0.143	↓-0.058	0.033	0.036
Mahottari	0.181	0.264	0.204	0.002	0.066	0.073

^a^4 districts—Mustang, Guimi, Nuwakot and Manang—have no raw coverage as no samples are located in those areas

^b^The RGI score measures relative inequality when given coverage levels. Negative values indicate a lower than expected inequality, while positive values indicate greater than expected inequality. ↓means a score significantly lower than 0, while ↑means significantly higher than 0

**Table 3:** District estimates for access to improved water and summaries of inequity coefficient

Districts	Model Estimate(JMP adj)	Raw coverage^a^	Gini	RGI score^b^	Theil *L*	Theil *T*
Bara	1.000	1.000	< 0.001	↓-0.014	< 0.001	< 0.001
Bardiya	1.000	0.995	0.002	↓-0.012	< 0.001	< 0.001
Dhading	1.000	0.987	0.027	↑0.013	0.009	0.008
Dhanusa	1.000	1.000	< 0.001	↓-0.014	< 0.001	< 0.001
Dolpa	1.000	1.000	0.012	−0.002	0.001	0.001
Gorkha	1.000	1.000	< 0.001	↓-0.013	< 0.001	< 0.001
Kailali	1.000	1.000	0.008	−0.005	0.001	0.001
Kapilbastu	1.000	1.000	0.008	−0.005	< 0.001	< 0.001
Mahottari	1.000	1.000	< 0.001	↓-0.013	< 0.001	< 0.001
Manang	1.000	–	< 0.001	↓-0.014	< 0.001	< 0.001
Morang	1.000	0.992	0.003	↓-0.010	< 0.001	< 0.001
Myagdi	1.000	0.902	0.004	↓-0.009	< 0.001	< 0.001
Parbat	1.000	1.000	0.001	↓-0.012	< 0.001	< 0.001
Parsa	1.000	1.000	< 0.001	↓-0.014	< 0.001	< 0.001
Rasuwa	1.000	0.987	< 0.001	↓-0.014	< 0.001	< 0.001
Rautahat	1.000	1.000	0.003	↓-0.010	< 0.001	< 0.001
Rupandehi	1.000	0.993	0.002	↓-0.011	< 0.001	< 0.001
Saptari	1.000	1.000	< 0.001	↓-0.013	< 0.001	< 0.001
Sarlahi	1.000	1.000	0.016	0.003	0.001	0.001
Siraha	1.000	1.000	0.001	↓-0.012	< 0.001	< 0.001
Sunsari	1.000	1.000	0.002	↓-0.011	< 0.001	< 0.001
Chitawan	0.999	0.960	0.013	−0.001	< 0.001	< 0.001
Nawalparasi	0.993	0.936	0.020	0.005	0.001	0.001
Nuwakot	0.991	–	0.028	↑0.012	0.004	0.004
Mustang	0.990	–	0.010	−0.006	< 0.001	< 0.001
Makwanpur	0.989	0.775	0.025	↑0.009	0.004	0.003
Syangja	0.987	1.000	0.019	0.003	0.001	0.001
Baglung	0.987	0.997	0.020	0.004	0.001	0.001
Rolpa	0.984	0.939	0.012	−0.005	< 0.001	< 0.001
Taplejung	0.979	0.985	0.016	−0.002	0.001	< 0.001
Jhapa	0.978	0.860	0.016	−0.002	< 0.001	< 0.001
Bhaktapur	0.969	0.963	0.015	−0.004	< 0.001	< 0.001
Kanchanpur	0.968	0.996	0.036	↑0.015	0.002	0.002
Solukhumbu	0.966	0.943	0.021	0.001	0.001	0.001
Udayapur	0.960	0.927	0.035	↑0.013	0.002	0.002
Kavrepalanchok	0.948	0.953	0.037	↑0.013	0.005	0.004
Lamjung	0.947	1.000	0.045	↑0.020	0.005	0.004
Banke	0.946	0.990	0.061	↑0.037	0.020	0.017
Khotang	0.945	1.000	0.029	0.005	0.002	0.002
Pyuthan	0.940	0.974	0.033	↑0.007	0.002	0.002
Sindhupalchok	0.930	0.887	0.018	↓-0.010	0.001	0.001
Rukum	0.915	0.985	0.050	↑0.019	0.005	0.004
Dolakha	0.915	0.880	0.022	↓-0.009	0.001	0.001
Baitadi	0.910	1.000	0.061	↑0.029	0.010	0.009
Darchula	0.909	0.919	0.040	↑0.008	0.003	0.003
Palpa	0.906	0.974	0.072	↑0.039	0.014	0.012
Ramechhap	0.866	0.849	0.028	↓-0.013	0.001	0.001
Kaski	0.862	0.866	0.025	↓-0.017	0.002	0.002
Sindhuli	0.858	0.645	0.065	↑0.022	0.008	0.007
Mugu	0.851	0.977	0.021	↓-0.023	0.001	0.001
Terhathum	0.839	0.869	0.036	↓-0.011	0.002	0.002
Dang	0.835	0.791	0.050	0.003	0.005	0.004
Humla	0.831	0.683	0.024	↓-0.024	0.001	0.001
Okhaldhunga	0.830	0.614	0.035	↓-0.013	0.002	0.002
Lalitpur	0.825	0.767	0.058	↑0.009	0.005	0.005
Ilam	0.823	0.600	0.038	↓-0.012	0.002	0.002
Panchthar	0.811	0.700	0.036	↓-0.016	0.002	0.002
Tanahu	0.805	0.809	0.089	↑0.035	0.012	0.012
Salyan	0.782	0.563	0.097	↑0.038	0.017	0.016
Jumla	0.765	0.725	0.044	↓-0.018	0.003	0.003
Dadeldhura	0.763	0.734	0.076	0.014	0.011	0.010
Dhankuta	0.740	0.716	0.044	↓-0.023	0.003	0.003
Bajura	0.739	0.904	0.089	↑0.021	0.013	0.012
Gulmi	0.710	–	0.123	↑0.050	0.024	0.023
Kathmandu	0.702	0.702	0.110	↑0.035	0.022	0.020
Bajhang	0.701	0.711	0.072	−0.003	0.008	0.008
Kalikot	0.669	0.704	0.053	↓-0.029	0.004	0.004
Surkhet	0.649	0.670	0.068	↓-0.017	0.007	0.007
Bhojpur	0.622	0.333	0.113	↑0.022	0.019	0.020
Jajarkot	0.622	0.256	0.088	−0.003	0.012	0.013
Arghakhanchi	0.616	0.380	0.126	↑0.033	0.024	0.024
Sankhuwasabha	0.613	0.563	0.137	↑0.044	0.029	0.029
Achham	0.581	0.535	0.079	↓-0.021	0.012	0.011
Dailekh	0.492	0.349	0.060	↓-0.058	0.006	0.006
Doti	0.417	0.340	0.103	↓-0.030	0.019	0.020

^a^4 districts—Mustang, Guimi, Nuwakot and Manang—have no raw coverage as no samples are located in those areas

^b^The RGI score measures relative inequality when given coverage levels. Negative values indicate a lower than expected inequality, while positive values indicate greater than expected inequality. ↓means a score significantly lower than 0, while ↑means significantly higher than 0

### Inequality analysis

**Fig. 4:**
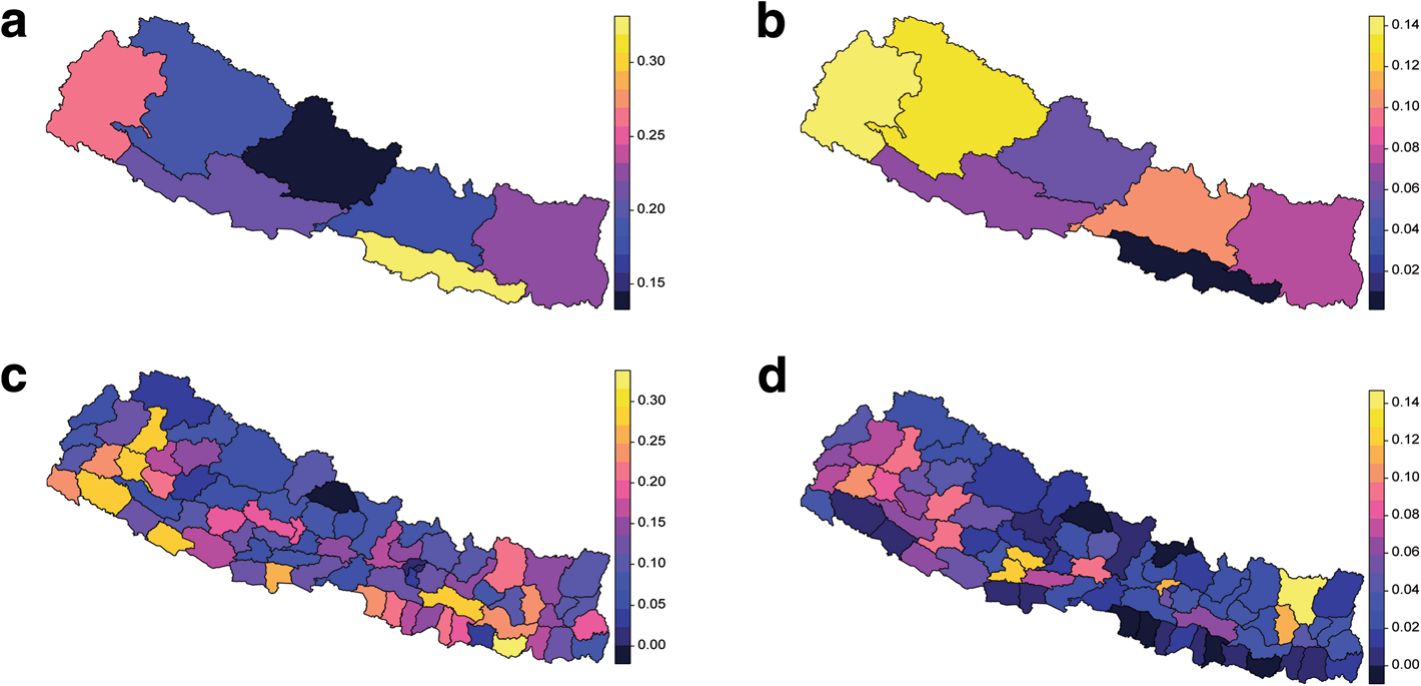
Geographic inequity (Gini coefficient) in access to WSS presented by province and district. Plots are shown for (**a**) improved sanitation by province, (**b**) improved drinking water by province, (**c**) improved sanitation by district and (**d**) improved drinking water by district

Although nationally inequality seemed not to overstep the warning limit of 0.4 for the Gini coefficient (Tables 1, 2 and 3), geographic inequality varied substantially in provinces and districts. Figure 4 shows the Gini coefficients for provinces and districts in the distribution of access to improved WSS. Nationally, inequality regarding improved sanitation was worse than that of improved drinking water: 0.272 in the Gini coefficient for improved sanitation (range: 0.145 in Province No.4 to 0.319 in Province No.2) versus 0.078 for improved drinking water (range: 0.136 in Province No.7 to 0.010 Province No.2). The most unequal districts with regard to improved sanitation were Saptari, Sindhuli, Banke, Bajura and Achham (ranging from 0.276 to 0.316), and Sankhuwasabha, Arghakhanchi, Gulmi, Bhojpur, Kathmandu (ranging from 0.110 to 0.137) regarding improved drinking water. The RGI score showed that 24 districts in improved sanitation and 24 districts in improved drinking water had a significantly higher than expected Gini coefficient when given coverage estimates.

**Fig. 5:**
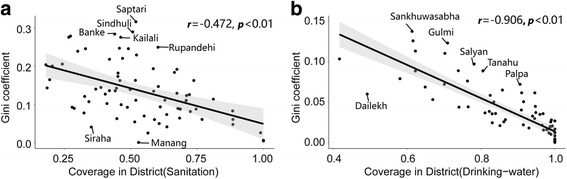
Correlation and regression between Gini coefficient and coverage of improved WSS by district. Plots are shown for (**a**) improved sanitation and (**b**) improved drinking water. Linear regression prediction with 95% confidence interval are shown in plots. Dots represent districts, and *r* is the Spearman pairwise correlation coefficient

Table 4 shows the decomposition of Theil *L* and Theil *T*, presented with within- and between-province inequality and within- and between- district inequality. First, there was consistently higher inequality in Theil *L* and Theil *T* terms among improved sanitation than among improved drinking water. Second, within-province inequality accounted for 72% or more of national inequality in the distribution of both improved sanitation and drinking water for both Theil *L* and Theil *T*. However, between-district inequality explained 60–70% of overall inequality. The Gini coefficient against coverage in districts reveals the significant inverse correlation, which was in line with other studies (*r* = − 0.472, *r* = − 0.960, *P* < 0.01 for sanitation and drinking water respectively). Figure 5 plots the result of a linear regression of the Gini coefficient on district coverage of improved WSS that was conducted, which identified outlier districts that had lower or higher levels of inequality than estimated given the level of coverage. We found many outliers in both improved WSS indicators. Several districts like Saptari, Sindhuli, Banke, Kailali and Rupandehi had higher than expected levels of inequality comparing the relationship between improved sanitation coverage and the Gini coefficient. The same pattern was observed for the districts Sankhuwasabha, Gulmi, Salyan, Tanahu and Palpa in the case of improved drinking water. There were also some apparent outliers with lower inequality than expected given the district coverage of improved WSS (for example: Dailekh for drinking water; Siraha and Manang for sanitation).

**Table 4:** Decomposition of overall inequity by province and district

Improved Indicator	Inequality Measure	Overall Inequality	Within-province inequality (% of overall)	Between-province inequality (% of overall)	Within-district inequality (% of overall)	Between-district inequality (% of overall)
Sanitation	Theil *L*	0.143	0.104(72.7%)	0.039(27.3%)	0.055(37.8%)	0.089(62.2%)
	Theil *T*	0.119	0.091(76.5%)	0.028(23.5%)	0.047(39.5%)	0.072(60.5%)
	Gini	0.272				
Drinking water	Theil *L*	0.017	0.015(88.2%)	0.002(11.8%)	0.005(29.4%)	0.012(70.6%)
	Theil *T*	0.015	0.014(93.3%)	0.001(6.7%)	0.005(33.3%)	0.010(66.7%)
	Gini	0.078

## Discussion

To our knowledge, this is the first attempt to comprehensively analyze and map the spatial heterogeneity and inequality of access to improved WSS conditions in Nepal at high resolution. Some provinces (sanitation: Province No.2; water: Province No.6) and districts (sanitation: Bajura, Achham, Doti, etc.; water: Arghakhanchi, Sankhuwasabha, Bhojpur, etc.) were identified as the most vulnerable according to the low coverage and degree of inequity. The efforts to reduce inequality should focus on the district level, as the between-district inequality was much greater than the within-district inequality. These results provide evidence to policymakers on future resource prioritization on increasing access to improved WSS, and thus may help eliminate and control NTDs such as lymphatic filariasis.

### Geographic differences in coverage of improved WSS

Consistent with previous studies [18], we found that geographical heterogeneity of improved sanitation was more severe than improved drinking water across Nepal. There was substantial geographical heterogeneity of improved sanitation across the nation between provinces and districts: Province No. 2 had the lowest coverage of 34.7%, while the adjacent Province No. 3 had the highest one of 79.3%. The difference may be attributed to the level of urbanization between those provinces. Data from the Global Rural Urban Mapping Project (GRUMP) [25] also suggest that the higher degree of urbanization is associated with higher coverage of improved sanitation. The results are consistent with other studies [39, 40]: in South Asia, urban residents are 2.2 times more likely to have access to an improved sanitation facility than rural residents [40]. In contrast, improved drinking-water sources are more related to environmental conditions. While having the lowest coverage in improved sanitation, Province No. 2 had the highest coverage for improved drinking-water sources, as it is mainly a lowland plain fed by the rivers from the north. Taking the terrain of Nepal into consideration [41], the Terai grasslands tended to have higher coverage of improved drinking-water sources when compared with the mountain and hill areas. The water supply system mostly exists in urban areas, and 83% of the Nepalese living outside of cities use natural sources such as streams and springs, and wells. The huge potential of water resources in streams, springs, wells, etc., ensures the sufficient supply of water, which might be the reason for better coverage in southern Terai than in the northern Hills or mountains. In general, the main reason for the geographical heterogeneity of improved sanitation and improved drinking-water sources might relate to the geographical heterogeneity of urbanization (finance, and human and technology resources) and water resources.

The areas with low coverage of improved WSS are likely impacted by environmental conditions and also by their socio-economic levels. It is thus crucial to develop affordable technologies and to increase investment to improve drinking-water sources and sanitation. In line with a policy framework recommended by the UN-Water Global Analysis and Assessment of Sanitation and Drinking-Water (GLAAS) [42], Nepal may 1) reinforce the implementation of specific plans for sustainable services for both water and sanitation, 2) develop a policy of testing water quality, 3) develop human resource strategies for WSS and attract skilled graduates to work in hygiene sectors and rural areas, and finally 4) increase funding to support rural areas and improved sanitation services. Furthermore, 24 districts (Ramechhap, Sindhuli, Bhojpur, Dhankuta, Sankhuwasabha, Terhathum, Ilam, Panchthar, Okhaldhunga, Baitadi, Dadeldhura, Achham, Bajhang, Bajura,Doti, Dailekh, Jajarkot, Jumla, Kalikot, Dang,Rukum, Salyan, Arghakhanchi,Gulmi) were lower than national coverage for both improved drinking water and improved sanitation, and thus deserve more attention in resource prioritization.

### Inequality in WSS distribution and its implications

Despite the increasing use of spatial analysis for health, few studies have focused on spatial inequality [43]. Our results revealed that better coverage of improved WSS is associated with less inequality in the same area which was consistent with previous studies [36]. Furthermore, both Theil *L* and Theil *T* suggested that within-province inequality was substantially greater than between-province inequality; while within-district inequality was obviously less than that of between-district. This indicates that policymakers should focus on reducing district-level inequality. Districts have long been the major administrative and governance entities in Nepal. Hence, our findings might also reflect the fact that long-standing administrative and governance differences between districts had the greatest influence regarding inequality and WSS. In the newly restructured federal Nepal, where local government authorities are even more decentralized, there will thus be further opportunities to address the inequity aspects of WSS at the district level. Though geographical inequality is associated with lower levels of coverage, many districts had higher than expected inequality (the same as the RGI score in Table 2 / Table 3) given the level of coverage (Fig. 5). Some researchers [44] suggest that policymakers should start with the areas with the lowest coverage and most inequality in order to achieve universal coverage.

### Implications of geographical heterogeneity and WSS coverage for disease prevention

Increasing focus on geographical heterogeneity and inequality of access to improved WSS is also vital for the prevention of epidemics [19, 20, 45]. Improved WSS plays an important role in preventing the diarrheal disease, water-borne diseases and especially (NTDs) like soil-transmitted helminthiasis, schistosomiasis or trachoma. Several studies have shown that populations with low coverage of WSS are also the ones which are substantially affected by NTDs [45]. Provision of safe water and adequate sanitation is one of five key public health strategies to control, eliminate or eradicate NTDs [46]. Regarding progress to eliminate lymphatic filariasis, Nepal has achieved 100% geographical coverage after multiple rounds of mass drug administrations (MDAs), but has not yet met the criteria of transmission assessment surveys (TAS) [47]. It is important to take into account several factors, including WSS, before implementing ambitious programs such as the elimination and control of NTDs like lymphatic filariasis [48–50]. The same holds true for other diseases like cholera that have a very strong relationship with geographic proximity and WSS status. We appeal to researchers and policymakers to obtain this greater disaggregation data to contribute to the elimination and control of NTDs or other water-borne diseases (see contact information above).

### Limitations

Despite our effort to minimize the challenges of working with limited data, we recognize that our estimation data on northern Nepal may have a greater level of uncertainty due to the availability of fewer clusters in this comparatively sparsely populated region. However, we believe that the sampling scheme based on the density of population and our adjustment by the JMP result have partially addressed that issue. Additionally, because of data availability, we did not include in our analysis additional covariates such as travel time, nightlight, and elevation which may reduce the robustness of our estimation. Lastly, the nature of this cross-sectional study prevented us from tracking patterns of inequality and heterogeneity over time. Future work should focus on change in these two elements.

## Conclusions

To our knowledge, this is the first study of its kind to explore geographical heterogeneity and inequality of access to improved drinking-water supply and sanitation across Nepal using a spatial distribution estimation with higher disaggregation than the administrative level. The study may facilitate prioritizing resource allocation and disease control to areas of greatest need with greater accuracy. This study found considerable geographical heterogeneity and inequality which were not shown in existing national statistics. Heterogeneity in access to improved WSS can be interpreted by geographical characteristics (mountains, hills, Terai and flowing river systems), population density and urbanization. Results showed that the main inequality is found between districts and within provinces. Therefore, district-level policy may be most suitable to addressing these inequity issues. Both heterogeneity and inequity in coverage of improved WSS need to be dealt with to protect the basic human right of the most vulnerable populations, to better control water-borne diseases, and to accelerate the achievement of SDGs. As considerable decision-making has been decentralized due to the recent administrative restructuring in Nepal, our findings may be particularly useful for policymakers and other stakeholders to address the issues of unequal access to reasonably improved WSS conditions, with the ultimate goal of universal coverage.

## Abbreviations

DHS: Demographic and Health Survey
GDP: Gross Domestic Product
GLAAS: Global Rural Urban Mapping Project
GRUMP: Global Rural Urban Mapping Project
JMP: Joint Monitoring Programme
MDGs: Millennium Development Goals
NTDs: neglected tropical diseases
RGI: Geographical Inequality Index
SDGs: Sustainable Development Goals
UN: United Nations
UNICEF: United Nations Children’s Fund
WHO: World Health Organization
WSS: Water Supply and Sanitation

## References

[1] Dattarao JV (2012). The human rights to safe drinking water and sanitation. APJMER.

[2] Stocks ME, Ogden S, Haddad D, Addiss DG, McGuire C, Freeman MC (2014). Effect of water, sanitation, and hygiene on the prevention of trachoma: a systematic review and meta-analysis. PLoS Med.

[3] Ziegelbauer K, Speich B, Mäusezahl D, Bos R, Keiser J, Utzinger J (2012). Effect of sanitation on soil-transmitted helminth infection: systematic review and meta-analysis. PLoS Med.

[4] Bartram J, Cairncross S (2010). Hygiene, sanitation, and water: forgotten foundations of health. PLoS Med.

[5] Norman G, Pedley S, Takkouche B (2010). Effects of sewerage on diarrhoea and enteric infections: a systematic review and meta-analysis. Lancet Infect Dis.

[6] Humphrey JH (2009). Child undernutrition, tropical enteropathy, toilets, and handwashing. Lancet.

[7] Esrey SA, Potash JB, Roberts L, Shiff C (1991). Effects of improved water supply and sanitation on ascariasis, diarrhoea, dracunculiasis, hookworm infection, schistosomiasis, and trachoma. B World Health Organ.

[8] Fund U N C (2013). Progress on sanitation and drinking water: 2015 update and MDG assessment.

[9] Mujica OJ, Haeberer M, Teague J, Santos-Burgoa C, Galvao LA (2015). Health inequalities by gradients of access to water and sanitation between countries in the Americas, 1990 and 2010. Am J Public Health.

[10] Narayanan R, van Norden H, Gosling L, Patkar A (2012). Equity and inclusion in sanitation and hygiene in South Asia: a regional synthesis. IDS Bull.

[11] Soares LCR, Griesinger MO, Dachs JNW, Bittner MA, Tavares S (2002). Inequities in access to and use of drinking water services in Latin America and the Caribbean. Am J Public Health.

[12] W.H.O, GLAAS, UN-Water Global Analysis and Assessment of Sanitation and Drinking-Water (2012). The challenge of extending and sustaining services.

[13] Pan A H O (2002). Health in the Americas: volume 1. J Epidemiol Community Health.

[14] Nepal Go (2012). National Population and housing census 2011.

[15] Bank TW (2013). World development indicators 2013. World Bank Publications.

[16] DeSA U (2013). World population prospects: the 2012 revision.

[17] Warner NR, Levy J, Harpp K, Farruggia F (2007). Drinking water quality in Nepal’s Kathmandu Valley: a survey and assessment of selected controlling site characteristics. Hydrogeol J.

[18] Fund UNCS (2015). Progress on sanitation and drinking water: 2015 update and MDG assessment.

[19] Aryal KK, Joshi HD, Dhimal M, Singh SP, Dhakal P, Dhimal B (2012). Environmental burden of diarrhoeal diseases due to unsafe water supply and poor sanitation coverage in Nepal. J Nepal Health Res Counc.

[20] Molyneux DH (2004). “Neglected” diseases but unrecognised successes-—challenges and opportunities for infectious disease control. Lancet.

[21] Boisson S, Engels D, Gordon BA, Medlicott KO, Neira MP, Montresor A (2016). Water, sanitation and hygiene for accelerating and sustaining progress on neglected tropical diseases: a global strategy 2015–20. Int Health.

[22] Shrestha A, Sharma S, Gerold J, Erismann S, Sagar S, Koju R (2017). Water quality, sanitation, and hygiene conditions in schools and households in Dolakha and Ramechhap districts, Nepal: results from a cross-sectional survey. Int J Env Res Pub He.

[23] Secretariat CA, Durbar S (2015). Constitution of Nepal 2015.

[24] ReliefWeb, Nepal Ecological Zone Map. In: ReliefWeb, 2000. http://reliefweb.int/sites/reliefweb.int/files/resources/7EFF22290C941713852577420057B80C-map.pdf. Accessed 28 Aug 2017.

[25] Balk D, Pozzi F, Yetman G, Deichmann U, Nelson A (2005). The distribution of people and the dimension of place: methodologies to improve the global estimation of urban extents. International Society for Photogrammetry and Remote Sensing, proceedings of the urban remote sensing conference.

[26] MOHP/Nepal, P (2011). Nepal Demographic and Health Survey 2011.

[27] Tatem AJ (2017). WorldPop, open data for spatial demography. Scientific Data.

[28] W.U.J.M (2004). Meeting the MDG drinking-water and sanitation target.

[29] UNICEF (2012). Progress on drinking water and sanitation.

[30] Larmarange J, Vallo R, Yaro S, Msellati P, Meda N. Methods for mapping regional trends of HIV prevalence from demographic and health surveys (DHS). Cybergeo: Europ. J Geo. 2011;558. 10.4000/cybergeo.24606.

[31] Larmarange J (2007). HIV prevalence in Africa : validity of a measurement.

[32] Wagstaff A, Paci P, Van Doorslaer E (1991). On the measurement of inequalities in health. Soc Sci Med.

[33] Theil H (1967). Economics and information theory.

[34] Yitzhaki S. Relative deprivation and the Gini coefficient. Q J Econ. 1979;93(2):321–4.

[35] Sen AK (1997). The welfare basis of real income comparisons: a survey. J Econ Lit.

[36] Pullan RL, Freeman MC, Gething PW, Brooker SJ (2014). Geographical inequalities in use of improved drinking water supply and sanitation across sub-Saharan Africa: mapping and spatial analysis of cross-sectional survey data. PLoS Med.

[37] Davies TM, Hazelton ML (2010). Adaptive kernel estimation of spatial relative risk. Stat Med.

[38] Larmarange J, Bendaud V (2014). HIV estimates at second subnational level from national population-based surveys. AIDS.

[39] Acharya A, Liu L, Li Q, Friberg IK (2013). Estimating the child health equity potential of improved sanitation in Nepal. BMC Public Health.

[40] Economic UNDo, Information UNDoP (2009). The millennium development goals report 2009: United Nations publications.

[41] Sharma CK (1977). Geology of Nepal. Educational enterprises.

[42] Water U, Organization WH (2015). Investing in water and sanitation: increasing access, reducing inequalities: GLAAS 2014 findings: highlights for the South-East Asia region.

[43] Anselmi L, Fernandes QF, Hanson K, Lagarde M (2013). Accounting for geographical inequalities in the assessment of equity in health care: a benefit incidence analysis. Lancet.

[44] Laxminarayan R, Chow J, Shahid-Salles SA (2006). Intervention cost-effectiveness: overview of main messages.

[45] Boisson S, Engels D, Gordon BA, Medlicott KO, Neira MP, Montresor A, et al. Water, sanitation and hygiene for accelerating and sustaining progress on neglected tropical diseases: a new Global Strategy 2015–20. Int Health 2016;8. http://apps.who.int/iris/bitstream/10665/182735/1/WHO_FWC_WSH_15.12_eng.pdf?ua=1. Accessed 28 Aug 2017. 10.1093/inthealth/ihv073 PMC5580794 26940305

[46] Organization WH. Investing to overcome the global impact of neglected tropical diseases. 2015;1(2):91–2. http://apps.who.int/iris/bitstream/10665/152781/1/9789241564861_eng.pdf?ua=1. Accessed 28 Aug 2017.

[47] Global programme to eliminate lymphatic filariasis: progress report, 2015. Wkly Epidemiol Rec. 2016 Sep 30;91(39):441–55. http://apps.who.int/iris/bitstream/handle/10665/250245/WER9139.pdf?sequence=1. Accessed 28 Aug 2017. 27758091

[48] Spiegel JM, Dharamsi S, Wasan KM, Yassi A, Singer B, Hotez PJ (2010). Which new approaches to tackling neglected tropical diseases show promise?. PLoS Med.

[49] Ngondi J, Gebre T, Shargie EB, Adamu L, Teferi T, Zerihun M (2010). Estimation of effects of community intervention with antibiotics, facial cleanliness, and environmental improvement (a, F, E) in five districts of Ethiopia hyperendemic for trachoma. Brit J Ophthalmol.

[50] Ngondi J, Matthews F, Reacher M, Baba S, Brayne C, Emerson P (2008). Associations between active trachoma and community intervention with antibiotics, facial cleanliness, and environmental improvement (a, F, E). PLoS Negl Trop Dis.

